# Genetic and morphological variation of *Vespa velutina nigrithorax* which is an invasive species in a mountainous area

**DOI:** 10.1038/s41598-022-08756-2

**Published:** 2022-03-18

**Authors:** Yuno Do, Woong-Bae Park, Jun-Kyu Park, Chang‐Jun Kim, Moon Bo Choi

**Affiliations:** 1 Department of Biological Sciences, Kongju National University, Gongju, Republic of Korea; 2 Research Panning and Coordination Team, Korea National Arboretum, Pocheon, Gyeonggi Republic of Korea; 3 Institute of Plant Medicine, Kyungpook National University, Daegu, Republic of Korea

**Keywords:** Ecology, Genetics, Zoology

## Abstract

The yellow-legged hornet (*Vespa velutina nigrithorax*) is an invasive species in South Korea with negative economic, ecological, and public health impacts. We investigated genetic and morphological variation in the species populations on Mt. Jiri, the tallest mountain in South Korea. We hypothesized that a high-altitude would be negatively correlated with the genetic diversity of the hornet population, and hornet wing morphology would change with an increase in altitude. Our results showed that the genetic diversity of yellow-legged hornets did not decrease as altitude increased. Regardless of the altitude, the inbreeding coefficient was high at the newly colonized sites. A single genetic population occurred in the mountainous areas examined and gradually expanded its range. Wing morphology, especially shape, did not change with an increase in altitude or decrease in temperature. Although snow cover and cool temperatures at high altitudes could limit nest-building activities, they did not prevent the extension of the range of the species. Therefore, the yellow-legged hornet cannot be controlled naturally by climate or topography; combined approaches, including chemical control, nest removal, and bait-trapping techniques should be implemented.

## Introduction

The yellow-legged hornet (*Vespa velutina nigrithorax*), native to China, was introduced in South Korea in 2003^1^ and it has rapidly colonized a large part of the country^2^. The spread of *V. velutina nigrithorax* has had negative economic, ecological, and public health effects^3–5^. However, approaches to control the species are still lacking and need to be studied continuously^6^.

High-altitude mountainous areas could serve as biogeographical barriers, as high altitudes may be negatively correlated with the occurrence of the yellow-legged hornet^7,8^. In particular, low-temperature conditions and snow cover in mountainous areas prevent the overwintering of mated queens. Nevertheless, the hornet is still found at high altitudes in mountain sites in South Korea. The country has a high proportion of mountainous areas when compared with other countries where the yellow-legged hornet successfully colonized, such as France, the UK, and Spain. Human-mediated transportation may explain the nest distribution at considerable distances from the invasion front; however, this dispersal may not be responsible for range expansion at high-altitude sites on mountains^9^. Nests of the yellow-legged hornet are more frequently observed at the edges of the mountains than at high-altitude sites; however, the hornet tends to gradually expand its range toward mountainous areas^4^. Investigating whether the yellow-legged hornet is genetically and morphologically responded to elevation gradients in mountainous ranges could facilitate the establishment of effective control strategies for this invasive species.

In this study, we examined genetic and morphological variations in yellow-legged hornet populations that have successfully colonized in the mountainous area in South Korea. We hypothesized that hornets at high-altitude sites have less genetic variation than hornets that have colonized low-altitude sites because those populations would have colonized the areas relatively recently, and the low temperatures and snow cover may interrupt overwintering and primary nesting after overwintering. This phenomenon can lead to isolated populations and low genetic diversity, which may destabilize the population and act as a mechanism to control the distribution or maintenance of the population^10–13^. In addition, we hypothesized that hornets that have colonized high-altitude sites have distinct wing morphology. Cold temperatures lower the wing beating frequency and thus power output, resulting in reduced flight performance. If flight is vital for colony maintenance, and wing loading increases at low temperatures, hornets could reduce wing loading by increasing wing size^14^. These morphological variations interact with the population structure and must be elucidated to understand the distribution of species.

## Results

### Genetic diversity and diversity indices among hornets

The overall allele frequency (N_A_) across seven loci and 39 yellow-legged hornet samples was 2.846 (2.49–3.286); the overall number of effective alleles (N_E_) was 2.444 (2.188–2.896); overall observed heterozygosity (H_O_) was 0.718 (0.524–0.857); overall expected heterozygosity (H_E_) was 0.667 (0.468–0.619); overall Shannon’s information index (I) was 0.920 (0.758–1.069); overall inbreeding coefficient relative to the population was 0.292 (− 0.529 to 0.121); and overall molecular diversity indices (number of different alleles, h) was 0.639 (0.562–0.743). In addition, overall inbreeding coefficient to the population was − 0.292 (− 0.419 to 0.121, Table 1).

**Table 1:** Genetic diversity and diversity indices of yellow-legged hornets (*Vespa velutina nigrithorax*) based on seven nuclear microsatellite loci estimated in 13 sampling sites: mean number of alleles (N_A_), number of effective alleles (N_E_), observed (H_O_) and expected (H_E_) heterozygosity, Shannon's information index (I), inbreeding coefficient relative to the population (F_IS_), and molecular diversity (h).

Group	N_a_	N_e_	H_o_	H_e_	I	F_IS_	h
G1	3.286	2.896	0.857	0.619	1.069	− 0.419	0.743
G2	2.857	2.365	0.619	0.54	0.903	− 0.203	0.562
G3	3.286	2.708	0.762	0.587	1.035	− 0.294	0.695
G4	2.571	2.188	0.762	0.532	0.838	− 0.45	0.648
G5	2.571	2.21	0.81	0.524	0.832	− 0.529	0.695
G6	2.429	2.19	0.619	0.468	0.758	− 0.329	0.686
G7	2.857	2.441	0.714	0.579	0.945	− 0.208	0.714
G8	2.714	2.39	0.619	0.54	0.882	− 0.116	0.61
G9	3	2.571	0.81	0.579	0.975	− 0.412	0.705
G10	2.857	2.42	0.714	0.571	0.933	− 0.199	0.648
G11	3	2.563	0.81	0.595	0.991	− 0.396	0.705
G12	2.571	2.363	0.714	0.508	0.826	− 0.36	0.638
G13	3	2.469	0.524	0.587	0.978	0.121	0.629
Overall	2.846	2.444	0.718	0.667	0.92	− 0.292	0.668

### Genetic distances and population structures

**Figure 1:**
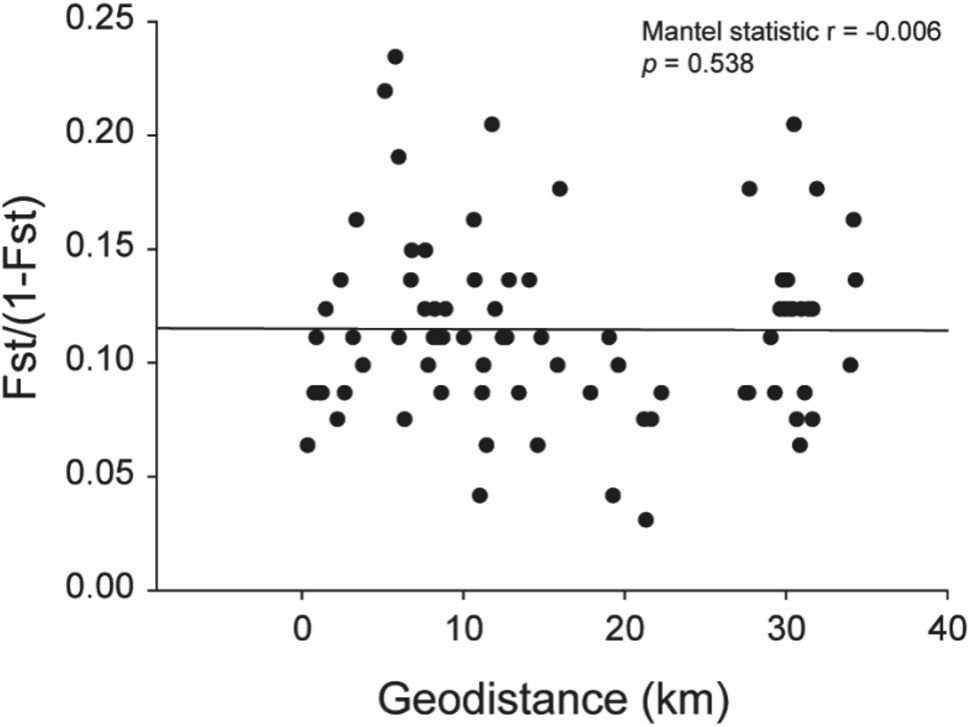
Correlation between the linearized pairwise F_ST_ and geographical distance for the 13 yellow-legged hornet sampling sites in Mt. Jiri. The Mantel test revealed no significant genetic isolation based on geographical distances among 13 sampling sites.

**Figure 2:**
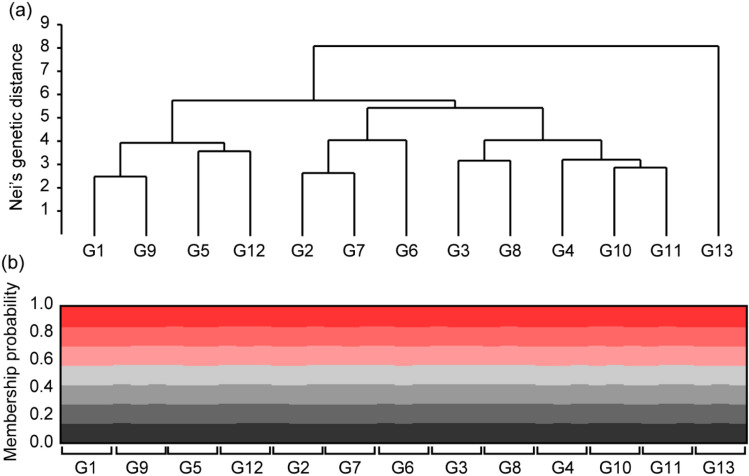
Results of Nei’s genetic distance and Bayesian clustering analysis in 39 yellow-legged hornets from 13 sites: (**a**) unweighted pair group method with arithmetic mean (Euclidean distance) tree among yellow-legged hornets from 13 sampling sites; (**b**) population clusters obtained by STRUCTURE analysis in yellow-legged hornets from 13 sampling sites.

**Figure 3:**
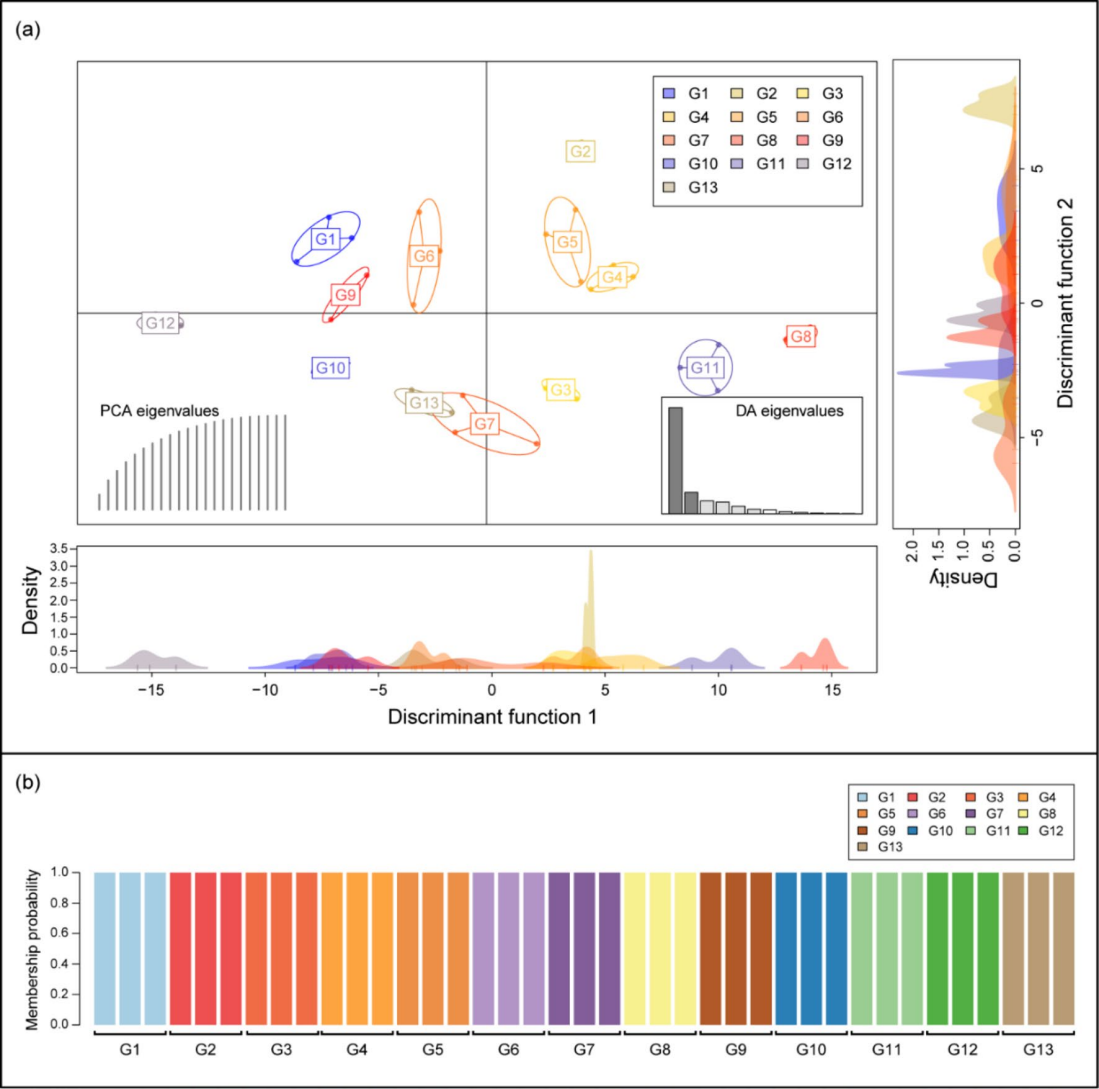
Results of population clustering by discriminant analysis of principal components (DAPC): (**a**) the discriminant functions 1 (DF1) explained 61.30% of the genetic variation in yellow-legged hornets from 13 sites and DF 2 explained 12.38% of the variation. Each node represents the genotype of a yellow-legged hornet connected to a centroid was assigned based on the clustering of the DAPC scores. (**b**) Membership probability of DAPC determined that the sampled individuals were optimally clustered into 13 groups.

The Mantel test showed no significant genetic isolation (Mantel statistic r =  − 0.006, *p* = 0.538) based on geographical distances among yellow-legged hornets from the 13 sampling sites (Fig. 1). According to the constructed unweighted pair-group method with arithmetic (UPGMA) tree, yellow-legged hornets from G13 were clearly separated from the other groups (Fig. 2a). However, the STRUCTURE analysis revealed that the population genetic structures of the yellow-legged hornets were not different among the 13 sampling sites. The 39 yellow-legged hornets from all sites were grouped into the same K-cluster (Fig. 2b). In Discriminant analysis of the principal components (DAPC), discriminant function 1 (DF1) explained 61.30%, and discriminant function 2 (DF2) explained 12.38% of the total genetic variation in the hornets. DF 1 indicated that yellow-legged hornets in G8, G11, and G12 were separated from the other groups, and the rest were grouped into three clusters (G1, G9, G10/G6, G7, G13/G2, G3, G4, and G5). Conversely, DF2 indicated that the hornets associated with G2 were isolated, whereas all the other groups were grouped into successive clusters (Fig. 3a). Finer-scale structures detected through DAPC separated the yellow-legged hornets from the 13 sites into 13 distinct groups (Fig. 3b).

### Morphological differences in wing shape

**Figure 4:**
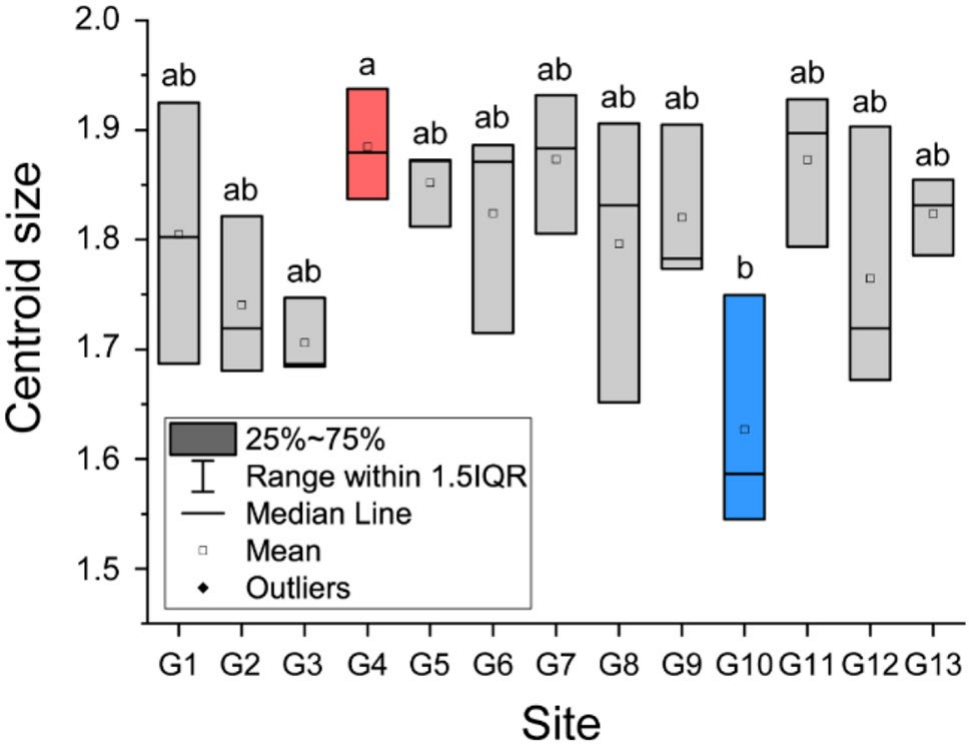
Comparison of centroid sizes in yellow-legged hornets based on landmark morphometrics analysis. The lines of the box graph represent the first quartile, median, and third quartile of the yellow-legged hornet. The transparent boxes represent average values. Lowercase letter and box color indicate differences (*p* < 0.05) in centroid size based on Tukey’s post-hoc tests.

Centroid size, which is a proxy for wing size, was significantly different (one-way analysis of variance (ANOVA), F = 2.304, *p* = 0.036) among the hornets from the 13 sampling sites (Fig. 4). Wing size in G4 was significantly larger (Tukey test, *p* < 0.05) than wing size in G10. There were no significant differences in wing size among the other groups.

**Figure 5:**
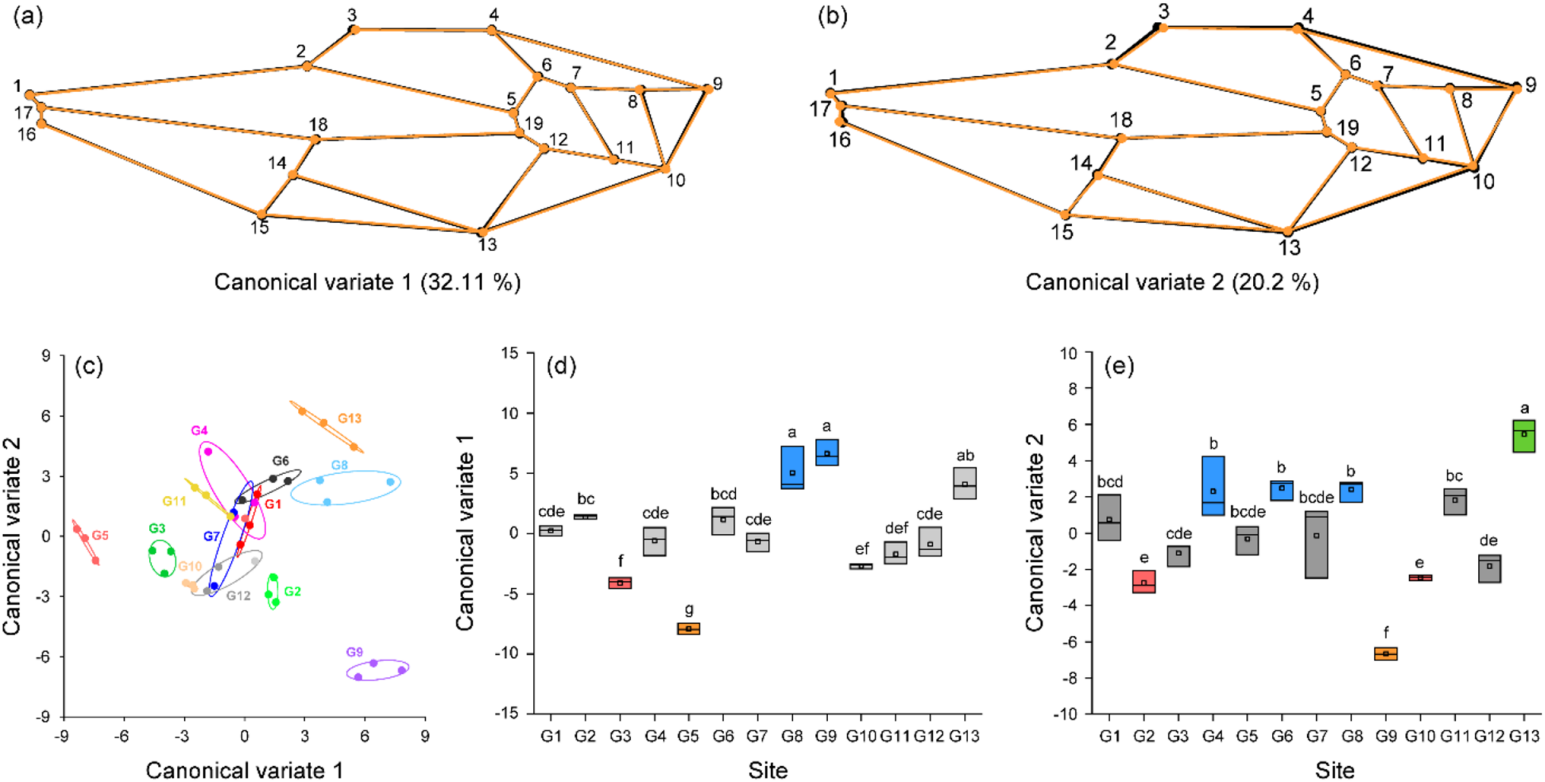
Canonical variate analysis (CVA) and wireframe graph with 19 landmarks illustrating morphological variance in wing shape among yellow-legged hornet from 13 sites: (**a**) wireframe graph illustrating minor morphological differences based on canonical variate 1 (CV1). (**b**) Wireframe graph illustrating minor morphological variation based on CV2. The wireframe of black circle and line represents shape with minimum CV value in CV wireframe graph, and the wireframe of orange circle and line represents shape with maximum CV value. Morphological variations in the major axes CV1 and CV2 were hardly detected. (**c**) Scatterplots of CV1 and CV2 from CVA of 19 landmarks for 39 yellow-legged hornets from 13 sites. (**d**) Comparison of CV1 values among yellow-legged hornets from the 13 sampling sites. (**e**) Comparison of CV2 values among yellow-legged hornets from the 13 sampling sites. Lowercase letter and box color indicate significant differences (*p* < 0.05) in CV1 and CV2 based on Tukey’s post hoc test.

Canonical variate analysis (CVA) results revealed two major morphological variations among the hornets from 13 sampling sites based on the two major axes CV1 and CV2 (Fig. 5a, b). CV1 explained 32.11% of wing morphological variance, and CV2 explained 20.20% of wing morphological variance. Changes in CV1 represent major variations in landmarks 3, 13, 8, and 9. As CV1 increases, the wing margin shape slightly changes. Changes in CV2 represent the major variations in landmarks 3, 4, 9, 10, 13, and 16. The shape of the wing slightly enlarges as CV2 increases. The wing shapes of hornets at each site were separated by CV1 and CV2 (Fig. 5c). CV1 (F = 45.22, *p* < 0.0001) and CV2 (F = 28.44, *p* < 0.0001) were significantly different among yellow-legged hornets from the 13 groups. Hornets in groups G3, G5, G8, and G9 were distinct (Tukey test, *p* < 0.05) from all the other groups based on CV1 (Fig. 5d). Based on CV2 (Tukey’s test, *p* < 0.05), hornets of groups at seven sites (G2, G4, G6, G8, G9, G10, G13) were separated from those of other groups (Fig. 5e).

The morphological distance is expressed based on Procrustes distance from CVA (Table 2). Unlike direct comparison of CV1 axis and CV2 axis, most of group did not have a significant morphological distance overall. Significant morphological distances were found only in some groups. Wing shape in G7 was significantly distant (*p* < 0.05) from that in G8. Wing shape in G10 was significantly distant (*p* < 0.05) from that in G11 and wing shape in G13 was significantly distant (*p* < 0.05) from those in G7 and G8.

**Table 2:**
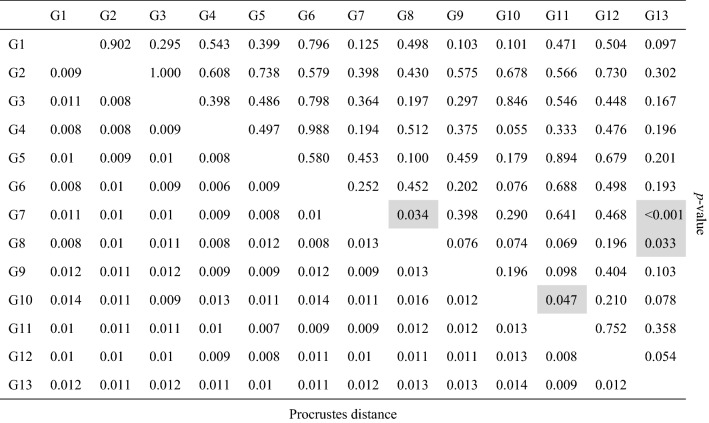
Morphological distances of yellow-legged hornets from 13 sampling sites.

Right value represents paired *p* values of Procrustes distances that were obtained using canonical variate analysis (CVA) after 10,000 permutation rounds among yellow-legged hornets from 13 sampling sites, and left value represents paired Procrustes distances from CVA. Significantly different distances are indicated by gray boxes.

## Discussion

The yellow-legged hornet is expanding its range in mountainous areas, regardless of altitude^2^. In the present study, adult hornets were collected at altitudes as high as 1100 m a.s.l. in mountainous areas. In Italy, adult hornets have been found at altitudes of 1200 m a.s.l, however, most nests of the yellow-legged hornet have been reported within 700 m a. s. l.^15^. The yellow-legged hornet can be observed at such high altitudes because its colony foraging radius is greater than 700 m^16^. Furthermore, in autumn, when foraging activity is the greatest, workers are found at sites relatively far from their nests^17^. Although snow cover and cool temperatures at high altitudes could limit hornet nest-building activities, Mt. Jiri, the highest mountain in South Korea, has not prevented the extension of the range of the yellow-legged hornet.

In this study, yellow-legged hornet genetic diversity did not decrease significantly with an increase in elevation. Yellow-legged hornet only exhibited a high inbreeding coefficient at a specific site. The high level of inbreeding is consistent with the genetic bottleneck experienced by hornets following their introduction in South Korea^18^. The genetic bottlenecks were not drastic enough to limit the expansion of the ranges of the yellow-legged hornet into mountainous areas. In addition, the hornets are likely to experience temporary genetic bottlenecks at the local level when expanding their colonies annually. In South Korea, there is a debate on whether the yellow-legged hornet invaded from a single or several sites^19^. In our study, although many hornets and nests were observed at relatively lower altitudes, their genetic structure did not show altitude-specific patterns. The yellow-legged hornet on Mt. Jiri appeared as a single population. The single genetic population is spreading at a slightly slower rate than in other countries, without being restricted by mountainous areas as geographical barriers.

Yellow-legged hornets that expand their ranges across or into mountainous areas may have no morphological adaptations. Although intraspecific minor variability in wing morphology may exist in the population investigated in the present study, wing morphology was not correlated with altitude. Additionally, yellow-legged hornets prefer cooler highlands and mountains in their original habitats^20^. A decrease in temperature due to an increase in altitude does not seem to be sufficient to alter wing shape. However, our study might be limited due to insufficient number of samples at high altitudes. We conducted permutation tests to resolve this limitation. Nevertheless, no altitude-specific pattern of morphological variation was observed. Our results demonstrated genetic and morphological adaptations specific to altitude in the hornets of Mt. Jiri; however, an extension of these results may be dangerous.

In conclusion, South Korea does not have a climate or topography that can naturally prevent the spread of wasps. In the early stages of the invasion of the yellow-legged hornet, the spread and establishment of the hornet in mountainous habitats was hampered by competition with an ecologically similar hornet, *Vespa simillima*^4^. However, *V. simillima*, which is less aggressive than the yellow-legged hornet, could have been forced out of the mountainous area, and the hornet occupied its place mountainous areas in South Korea^4^. Typically, nest removal and bait-trapping are used to control the yellow-legged hornet. However, eradicating and controlling hornets that occupy large mountainous areas using such techniques is challenging and impractical^21^. Therefore, chemical control techniques, including the use of insect growth regulators that have not been considered so far, could be adopted in mountainous areas, which is a core habitat of the yellow-legged hornet in South Korea.

## Material and methods

### Sampling sites

**Figure 6:**
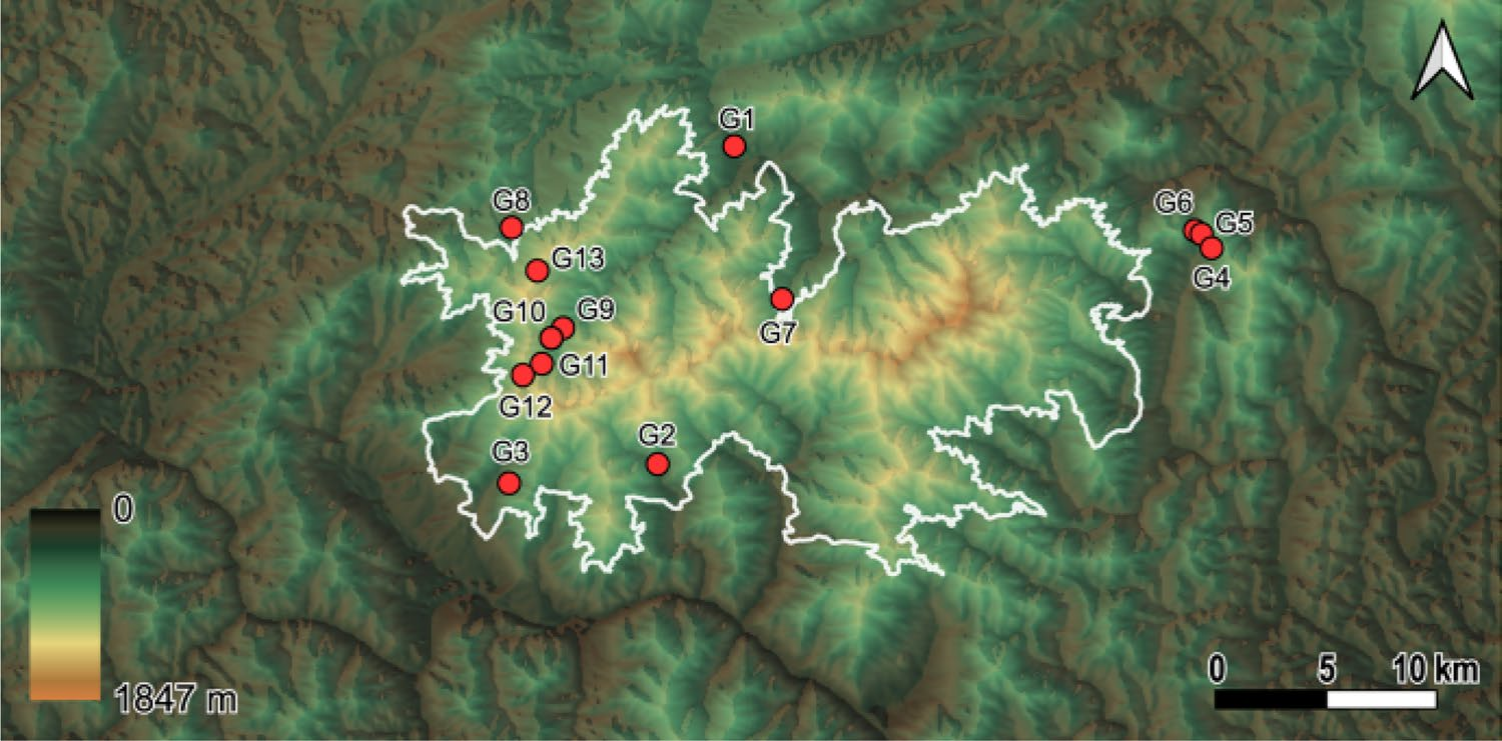
Thirteen yellow-legged hornet (*Vespa velutina nigrithorax*) sampling sites in Mt. Jiri National Park, South Korea. White line represents the boundary of Mt. Jiri National Park. The colors on the map represent elevation.

We collected yellow-legged hornets using traps at Mt. Jiri National Park in September 2019 when the colony was at its maximum size and the production of new queens resulted in high demand for hornet workers. At a lower altitude (approximately 200–800 m) and a higher altitude (approximately 800–1200 m), a total of 20 and 3 hornet nests were identified, respectively. Sugared water with acetic acid and 95% ethanol was used as the trap solution in 2 L plastic containers. The traps were monitored every two weeks. The collected hornets were preserved in 95% ethanol for use in later molecular and morphological analyses. Same individuals were used in both genetic and morphological analysis, for tracking response of individual units in two types of variations. Traps were placed in 30 sites and hornets were captured from 13 sites (Fig. 6, Table 3). We selected three individuals from each site, totalizing 39 similar-sized workers.

**Table 3:** Sample size, elevations and temperatures of 13 sampling sites where yellow-legged hornets (*Vespa velutina nigrithorax*) were collected.

Group	N	Altitude	Temperature
G1	3	385	13.2
G2	3	437	12.7
G3	3	523	12.1
G4	3	526	12.1
G5	3	542	11.8
G6	3	583	11.6
G7	3	650	11.1
G8	3	660	10.9
G9	3	726	10.5
G10	3	848	9.9
G11	3	962	9.4
G12	3	1042	8.9
G13	3	1185	8.1
Mean	3	697	10.9

Avg. temperature indicates the average annual temperature in 2020 at the sites, measured by the National Park Office of Jiri Mt.

### DNA extraction and microsatellite genotyping

We collected muscle samples from the hind legs of each worker for genomic DNA extraction. DNeasy Blood and Tissue kits (Qiagen, Hilden, Germany) were used to extract genomic DNA according to the manufacturer’s protocol. We measured the concentrations and quality of genomic DNA using a NanoDrop 2000 spectrophotometer (Thermo Scientific, Wilmington, USA), and the concentrations of genomic DNA were diluted between 10 and 20 ng/µL range. Seven polymorphic microsatellite loci were amplified using primers developed by^22^ for the yellow-legged hornet (D2-185, D3-15, R1-137, R1-36, R4-33, R1-75, and R1-169). We performed amplifications using the procedures for each locus detailed in^22^. Seq-Studio Genetic Analyzer (Applied Biosystems, Foster City, USA) was used to visualize the amplicons, and GeneMapper version 6.0 was used to evaluate the dataset for genotype errors and the presence of null alleles.

### Analysis of genetic diversity and distance, and population structure

**Figure 7:**
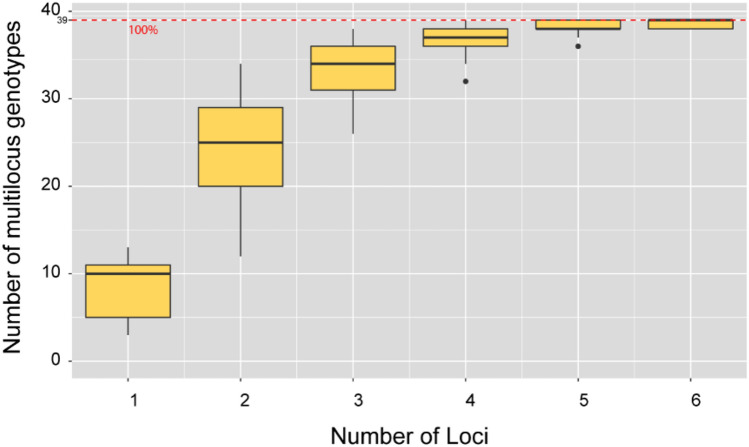
Number of multilocus genotypes based on number of loci from 39 individual of yellow-legged hornet. When the number of loci was 6 or more, the 39 individuals could be separated about 100%.

The genotype datasets were used to analyze genetic diversity and population structure. GENEPOP version 4.7^23^ was used to confirm deviations in Hardy–Weinberg equilibrium (HWE) and linkage disequilibrium of seven microsatellite loci. Null alleles, significant deviations from HWE, or evidence of linkage disequilibrium were not observed in any of the seven loci. In addition, seven loci could sufficiently separate 39 samples of yellow-legged hornets (Fig. 7).

The MS Excel add-in, GenAlEx version 6.5^24^, and Arlequin version 3.5^25^, were used to calculate genetic diversity and diversity indices, namely mean number of alleles (N_A_), effective number of alleles (N_E_), observed heterozygosity (H_O_), expected heterozygosity (H_E_), Shannon’s information index (I), molecular diversity (h), and inbreeding coefficient relative to the sub-population (F_IS_) of each population. The UPGMA based on Euclidean distances was used to construct a dendrogram based on Nei’s genetic distances obtained using GenAlEx version 6.5, using PAST 3^26^. Paired population differentiation (F_ST_) was performed using Arlequin version 3.5. The linearized F_ST_ value (F_ST_/[1 − F_ST_]) with geographic distance was used in the Mantel test (number of permutations: 999) to find evidence of genetic isolation by distance from the hornet using the ‘vegan’ package in R^27^.

**Figure 8:**
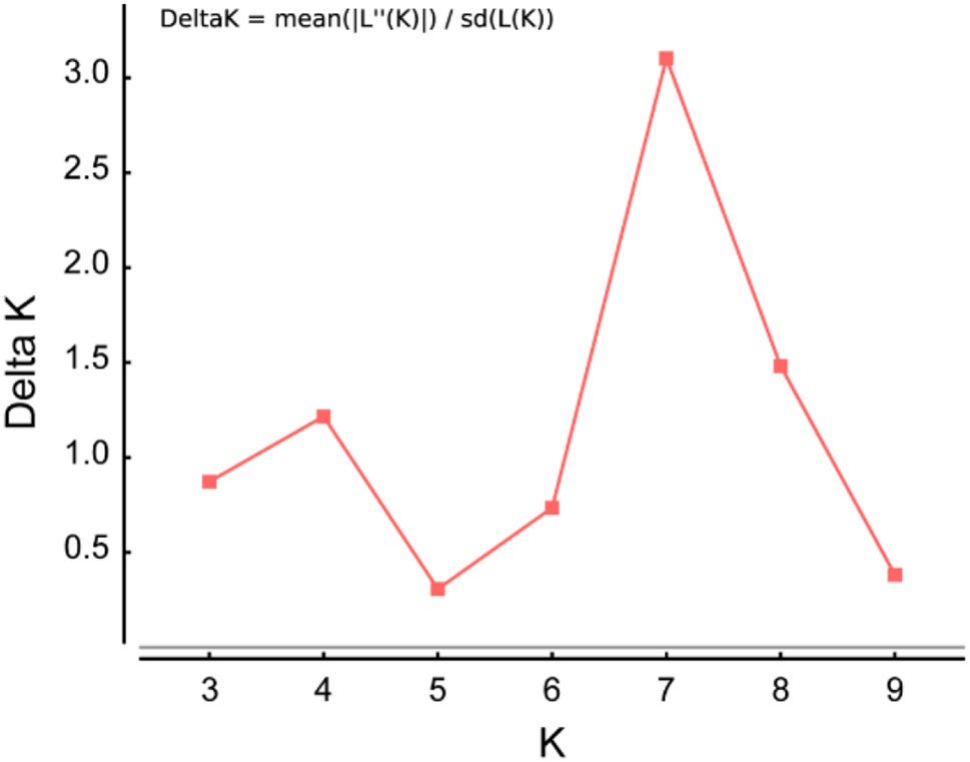
Optimal k-means for STRUCTURE analysis obtained by the ΔK (delta K) method in STRUCTURE harvest. Here, 100,000 simulations after burn-in of 100,000 simulations were used to obtain the three independent runs for range of 2 to 14 possible clusters. The highest delta K value was 7 (3.102).

Bayesian clustering was performed using STRUCTURE version 2.3.4^28^. STRUCTURE is an admixture model, which allows us to confirm whether ancestors in population k have passed a portion of their genetic material to individual *i*. Simulations (100,000) were performed in each analysis after an initial burn-in of 100,000 simulations. We used the ΔK method^29^ in STRUCTURE Harvester^30^, which supported the estimation of the best‐identified k value. The range of one to 14 possible clusters with three independent runs each was employed in STRUCTURE Harvester. In the STRUCTURE harvest analysis results, the best-supported K value among hornets across 13 populations was identified as 7 (Fig. 8).

DAPC^31^, which is a multivariate clustering algorithm, was used to analyze the population structures of 39 hornets from 13 sites using the “adegenet” package in R^32^. The analysis describes the greatest amount of variation through a discriminant function representing a linear combination of correlated alleles in a linear discriminant analysis based on principal components generated after reducing the dimension of the genetic variation using principal component analysis^33^. The method was used to determine how distinct each population among the 13 sites was.

### Morphological analysis

**Figure 9:**
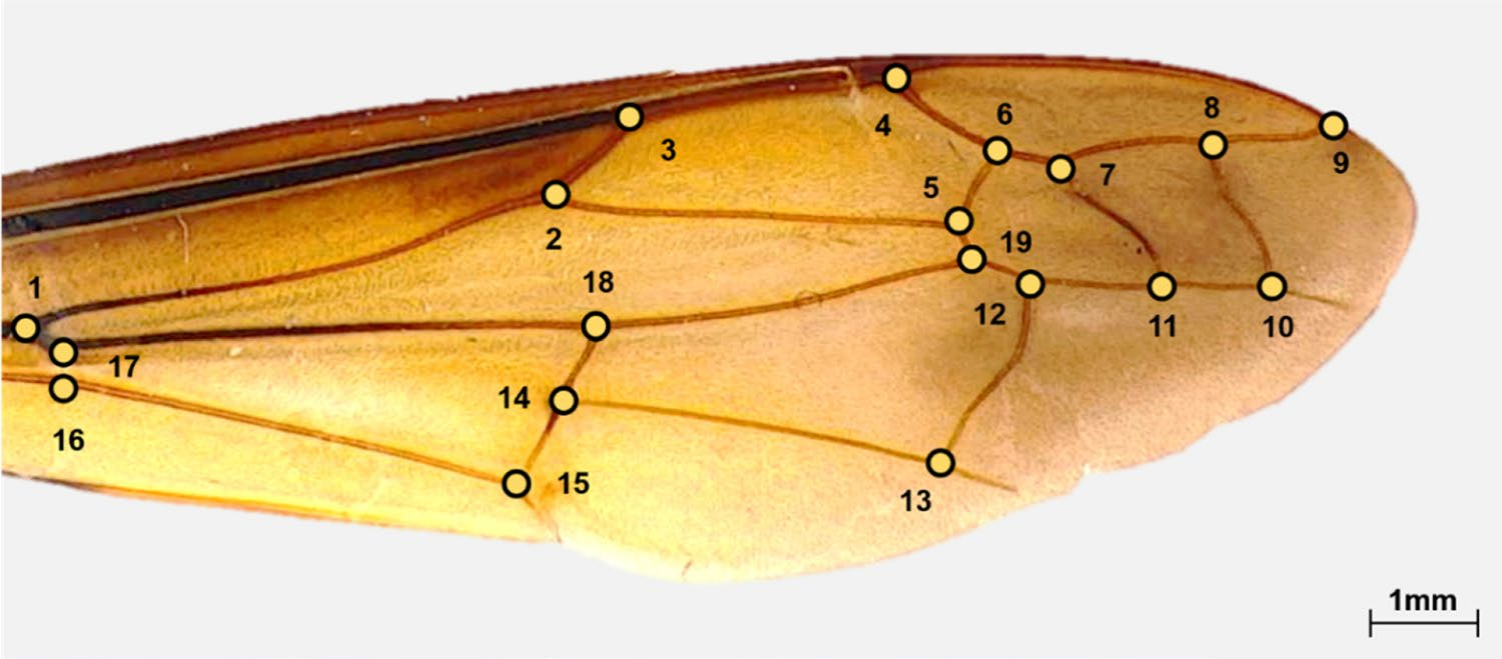
Locations of the landmark coordinates in the right forewings of a yellow-legged hornet. Nineteen landmarks were designated in the wing vein.

Landmark-based geometric morphometrics were used to analyze the wing shapes of the hornets. TpsDig^34^ was used to digitize the 19 landmark points in the wing vein (Fig. 9). Morpho J version 1.07a (Manchester, UK) was used to convert the digitized landmark coordinates into Procrustes coordinates. Centroid size, which used the size proxy in landmark morphometrics^35^, was measured to compare wing sizes among the 13 groups. CVA was used to compare morphological differences among the 13 groups and to calculate the f contribution (%) of each canonical variate (CV) in CVA. Variation in wing shape among the hornets from 13 sites was visualized in a wireframe graph along the first two (CV1 and CV2) axes. We also identified the values and significant distances of the Procrustes from CVA to explore the morphological similarity between groups. Significant morphological distances were obtained using CVA after 10,000 permutation rounds. One-way ANOVA was used to test significant differences in centroid size, CV1, and CV2, among the hornets using GraphPad Prism version 7.0 for Windows (GraphPad Software, San Diego, USA). When significant differences were found in the ANOVA test, Tukey’s post hoc tests were performed. All differences were considered significant at *p* < 0.05.
